# Biochar Derived from Post-Adsorbent for Immobilizing Cu and Cd in Sediment: The Effect on Heavy Metal Species and the Microbial Community Composition

**DOI:** 10.3390/toxics11080666

**Published:** 2023-08-02

**Authors:** Qinju Sun, Shaohua Lin, Guohua Liu, Pingping Li

**Affiliations:** 1 College of Biology and the Environment, Nanjing Forestry University, Nanjing 210037, China; qinjusun@njfu.edu.cn (Q.S.);; 2 School of Civil Engineering, Nanjing Forestry University, Nanjing 210037, China; 3 Bamboo Research Institute, Nanjing Forestry University, Nanjing 210037, China; ghliu@njfu.edu.cn

**Keywords:** biochar, post-adsorbent, sediment remediation, heavy metal, enzymes activity, microbial community composition

## Abstract

Many biomass wastes or their modified forms have been investigated as heavy metal adsorbents. However, less emphasis has been placed on post-adsorbent management or possible further utilization. In this study, biochar (BC) derived from modified bamboo adsorbent after the adsorption of Cu from an aqueous solution was used for the in situ remediation of lake sediment contaminated with Cd and Cu. The results indicated that the Cu concentration was extremely low (≤0.015 mg/L), while Cd was not detected in the overlying water or the interstitial water after the 90-day BC treatment. The pH value (7.5–8.1) slightly increased, and the toxicity characteristic leaching procedure (TCLP) leachability of the Cu and Cd in the sediment decreased overall. Cu and Cd were preferentially transformed into more stable species. The findings highlighted the potential possibility of BC derived from post-adsorbent being used for sediment remediation. However, the BC addition produced significant effects on the sediment microbial activity and community structure. In general, with an increase in BC, the urease activity increased, while the alkaline phosphatase and invertase activity decreased, which could be attributed to the BC itself. In addition, significant changes in both bacterial and fungal genera were observed. Hence, a cautious approach should be taken in the practical application of BC.

## 1. Introduction

Heavy metal pollution has non-degradable and bio-accumulative properties, which may result in huge threats to environmental ecosystems and public health [1,2,3]. Adsorption is regarded as a facile and efficient method to remove heavy metals from polluted water; therefore, much effort was made to prepare adsorbents with high adsorption capacities [4,5]. Diverse adsorbents were used to remove heavy metals from water, such as charcoal, activated carbon, zeolites, polymers, and biomaterials [6,7,8,9].

Environmentally friendly and readily available biomaterials from agricultural wastes and forestry represent good sources of cost-effective adsorbents, containing numerous functional groups—such as carboxyl, carbonyl, amine, hydroxyl, and ether groups—that can act as ligands for metal ion complexation in aqueous media [10,11]. Furthermore, new functional groups are easy to be introduced onto lignin and cellulose, the primary components of these materials, through hydroxyl reactions, to develop heavy metal adsorbents [12,13]. Many agricultural wastes and forestry materials or their modified forms were investigated as heavy metal adsorbents, including fruit peels, tea wastes, sawdust, coconut husk, and bamboo [12,14,15,16,17,18].

Most research was dedicated to finding or developing new biomass adsorbents, whereas less emphasis was placed on post-adsorbent management or possible further utilization. Improper disposal, such as incineration and landfills, may produce secondary pollution [10]. In recent years, researchers have paid increasing attention to the development of sustainable technologies to minimize the cost of materials/techniques and to more efficiently utilize resources, and research reports have shown positive prospects for the upcycling or use of post-adsorbents [10,19]. Biomass post-adsorbents for heavy metal removal were successfully explored as catalysts [20], fertilizers, soil conditioners [21], and bioactive compounds [22].

In an aquatic environment, heavy metals that preferentially associate with surface sediments can enter the overlying water once sediment resuspension occurs, which may further impact local water quality and ecological safety [23]. Moreover, the remediation of heavy-metal-contaminated sediment has gradually become a major worldwide concern. A successful immobilization remediation technique must cost-effectively improve the stabilization of heavy metals in sediments by reducing their mobility, bioavailability, and toxicity [24,25]. The lower cost and more efficient in situ sediment remediation technologies are increasing in popularity over ex situ remediation [26]. Biochar (BC) and modified BC, due to the wide availability of their raw materials, low cost, and favorable physical/chemical characteristics, have been increasingly studied for the in situ remediation of contaminated sediment [11,27,28,29]. Some studies demonstrated the excellent potential of BC to reduce or immobilize heavy metals (Cu^2+^, Pb^2+^, Cd^2+^, Zn^2+^, and Hg^2+^) in contaminated sediments [26].

It is worth noting that the application of BC may produce two conflicting effects on heavy metal mobility and bioavailability: immobilizing heavy metals to reduce bioavailability or mobilizing heavy metals to increase bioavailability [30]. Furthermore, BC applications can also pose potential ecological risks. Studies showed that enzyme activities and the indigenous microbial community can be affected after BC application [31,32,33].

BC derived from different feedstocks typically has different properties, and these may have different effects or impacts on the mobility of heavy metals and microbes. However, to the best of our knowledge, little was reported on the feasibility or risk of the potential application of BC produced from post-adsorbents for sediment remediation, especially when the BC contains a certain amount of metal elements. In this study, BC was derived from modified bamboo adsorbent after the adsorption of Cu from an aqueous solution and then used for the in situ remediation of Cd- and Cu-contaminated lake sediment. The objectives of this study were to (1) investigate the effectiveness of this BC on the Cd and Cu concentrations in sediment interstitial waters and the sediment overlying the water; (2) investigate the effectiveness of the BC on Cd and Cu immobilization in the sediment; and (3) study the effect on the enzyme activities and microbial community due to the BC application.

## 2. Materials and Methods

### 2.1. Preparation of the Post-Adsorbent and BC

The adsorbent was prepared as follows. The culm waste of moso bamboo was cut into blocks, washed using deionized water, dried at 105 ± 2 °C to a constant weight, ground into a powder, and sieved to obtain a powder size between 60 and 100 mesh. After 3 g of the powder were mercerized with 500 mL of 2 mol/L NaOH solution, the mercerized bamboo powder was mixed with 100 mL of 8% NaOH and 15 mL of epichlorohydrin in a three-necked flask at 40 °C for 8 h for epoxidation. The epoxidation product was then thoroughly washed with absolute ethyl alcohol and deionized water. In the next step, in order to obtain amine-group-functionalized adsorbent, 5 mL of tetraethylenepentamine was added dropwise into the flask with 100 mL of 1% Na_2_CO_3_ at 40 °C for 2 h. The final product was thoroughly washed with absolute ethyl alcohol and deionized water and dried at 60 °C.

The adsorption experiment was conducted as follows. A total of 1.0 g of adsorbent was added to 25 mL of a working solution with a Cu(II) concentration of 40 mg/L at pH 6.0. This was then shaken at 150 rpm for 24 h to reach equilibrium. After drying, the post-adsorbent was converted into BC using a tube resistance furnace with a limited oxygen environment using high purity nitrogen (>99.999%) at a flow rate of 100 mL/min as protective gas. The heating temperature was increased from the ambient temperature to 600 °C at a heating rate of 10 °C/min and held at the final temperature for 1 h. The temperature was then dropped to room temperature to obtain the BC loaded with Cu. Selected properties of the BC are shown in Table 1. The scanning electron microscope (SEM) and Fourier-transform infrared spectroscopy (FTIR) results are shown in Figure S1. The BC sample had an underdeveloped pore structure that explained the low specific surface area. However, the BC sample contained reactive functional groups, such as carboxyl and hydroxyl groups, as shown in the FTIR spectrum.

### 2.2. Sediment–BC Contact

Sediment (approximate 15 cm depth) was collected from Xuanwu Lake (32°04′05″ N, 118°48′19″ E), Nanjing, China, using a Peterson grab. The collected sediment was air-dried, crushed, and ground through a 100-mesh sieve. Then, 900 g sediment was mixed with 720 mL of 125 mg/L CuSO_4_ and 180 mL of 37 mg/L CdSO_4_ solution and pre-incubated with a water content of 50% at 20 °C for 30 days. After the pre-incubation was finished, the Cu- and Cd-rich sediment was air-dried in a cool and ventilated place, ground through a 100-mesh sieve, and stored in a self-sealing bag at 4 °C for use. The sediment properties are shown in Table 2.

After weighing 40 g of the air-dried sediment into 250 mL screw bottles, 200 mL of deionized water was added to the bottles. When the sediment was incubated and stabilized for 7 days, 0% (CK), 1% (0.4 g), 2% (0.8 g), and 5% (2 g) of BC was added to the Cu- and Cd-contaminated sediment bottles (three replicates were established for each treatment), and the BC and sediment were mixed and incubated in screw bottles in an artificial climate box at 20 °C for 90 days in the dark.

### 2.3. Cu and Cd Contents in the Water and Sediment

After 90 days of incubation, the overlying water was sampled and filtered through a 0.22-μm aqueous phase needle filter. The sediment samples were centrifuged at 4000 rpm for 15 min, and the separated interstitial water was obtained and then filtered through a 0.22-μm aqueous phase needle filter. A portion of each centrifuged sediment was air-dried in a cool and ventilated place, crushed, and sieved through a 100-mesh sieve for the different fractions and mobility analyses of the heavy metals and the enzyme activities assay. The concentrations of the Cu and Cd heavy metals in the overlying water and interstitial water were measured using atomic adsorption spectroscopy (TAS-990 Super F, Beijing Pursee General Instrument Co., Ltd., Beijing, China). The rest of the sediment was stored at 4 °C for further microbial community determination.

The total heavy metal concentrations in the sediment were extracted using a tri-acid digestion method. A total of 0.1 g of dry sediment sample in a 30 mL polytetrafluoroethylene (PTFE) crucible was heated on an electric hot plate. The heating procedure consisted of three stages: the first stage of the 5 mL HNO_3_ digestion consisted of heating at a constant temperature of 150 °C for 15 min; the second stage of the 5 mL HF digestion consisted of heating at 150 °C for 20 min; and the third stage of the 3 HClO_4_ digestion consisted of heating at temperature 240 °C until white smog was out. The digested sample was then dissolved using 5 mL of a mixed acid of HNO_3_-HCl (volume ratio 20:1; diluted twice with DI water; the final concentrations of these two acids were 32.3% and 0.9%, respectively), which was transferred to a 100 mL capacity bottle to be measured using atomic adsorption spectroscopy.

### 2.4. Sequential Extraction Procedure and Toxicity Characteristic Leaching Procedure

The different fraction of heavy metals in the sediment samples were determined using the BCR extraction [24,33]. Four different fractions of Cu and Cd were extracted as follows. (1) The acid-soluble fraction (F1) was extracted with 0.11 mol/L CH_3_COOH. A mixture of 1 g of the sample and 40 mL of CH_3_COOH was shaken at 250 rpm for 16 h. (2) The reducible fraction (F2) was extracted with 0.5 mol/L NH_2_OH∙HCl. The residue from step 1 with 40 mL NH_2_OH∙HCl was shaken at 250 rpm for 16 h. (3) The oxidizable fraction (F3) was extracted by 8.8 mol/L H_2_O_2_ and 1.0 mol/L CH_3_COONH_4_ (adjusted to pH 2 with HNO_3_). The residue from step 2 with 10 mL H_2_O_2_ was heated at 85 °C until nearly dry, and then the residue with 25 mL CH_3_COONH_4_ was shaken at 250 rpm for 18 h. (4) The residual fraction (F4) was extracted using the tri-acid digestion method (HNO_3_, HF, and HClO_4_). The extracted fluid was filtered through a 0.22-μm aqueous phase needle filter before the filtrate was analyzed for Cu and Cd using atomic adsorption spectroscopy.

The toxicity characteristic leaching procedure (TCLP) was also conducted following US EPA Method 1311 to evaluate the mobility of heavy metals in the samples. The extraction fluid was prepared by diluting 5.7 mL CH_3_COOH with 500 mL ultrapure water before adding 64.3 mL NaOH (1 mol/L) to a volume of 1000 mL using 1 mol/L HNO_3_ to adjust the pH = 4.9 ± 0.1. A total of 2 g of sediment was then mixed with 40 mL of the extraction fluid in a 50 mL centrifuge tube and shaken (190 rpm) for 19 h at 25 °C. After the tube was centrifuged at 4000 r/min for 15 min, the supernatant was filtered through a 0.22-μm aqueous phase needle filter for subsequent analysis.

### 2.5. Determination of Enzymes’ Activities and the Microbial Community

Three enzymes (urease, alkaline phosphatase, and invertase) were analyzed using the colorimetric method [31]. The urease activity was assayed by determining the ammonium released from a solution of urea (10%) and citrate buffer (pH 7) after being incubated at 37 °C for 24 h. The ammonium content was determined at 578 nm using a spectrophotometer, and the urease activity was expressed as μg of ammonium per gram of sediment (dry weight). The alkaline phosphatase activity was measured by the transformation of disodium phenyl phosphate to phenol. The phenol content was determined at 510 nm, and the alkaline phosphatase activity was expressed as μmol of phenol per gram of sediment (dry weight). The invertase activity was assayed based on the glucose product using a sucrose solution as the substrate. The glucose product was colorimetrically determined at 508 nm and expressed as mg of glucose per gram of sediment (dry weight).

DNA in the sediment samples was extracted using the E. Z. N. ATM Mag-Bind Soil DNA Kit (Omega Bio-tech (Shanghai) Co., Ltd, Shanghai, China). The concentration and purity of DNA were monitored on agarose gels. The products were stored at −20 °C until further analysis. The V3–V4 region of the bacterial 16S rRNA was amplified using the primer set 338F (5′-ACTCCTACGGGAGGCAGCA-3′) and 806R (5′-GGACTACHVGGGTWTCTAAT-3′). The fungal internal transcribed spacer (ITS) region of the rRNA gene copies was amplified using the primer set ITS1F (5′-CTTGGTCATTTAGAGGAAGTAA-3′). After the amplification and subsequent purification, the DNA samples were sequenced using NovaSeqPE250 sequencing technology. After quality filtering, the bacterial and fungal sequences were aligned with those archived in the QIIME2 dada2 and UNITE ITS databases, respectively, and clustered into operational taxonomic units (OTUs) at an identity level of 97%. The diversity (Shannon and Simpson indices) and richness (Chao1) were calculated for the bacteria and fungi based on the taxonomic information.

### 2.6. Statistical Analysis

The treatment effects (0%, 1%, 2%, and 5% BC) on sediment pH, the concentrations of TCLP of Cu and Cd, and three enzymes were evaluated using a one-way analysis of variance (ANOVA) with repeated measures. The above statistical analyses were performed using SPSS software (version 22.0, IBM Inc., Armonk, NY, USA).

## 3. Results and Discussion

### 3.1. Cu and Cd Concentration in the Sediment Water Environment

**Figure 1:**
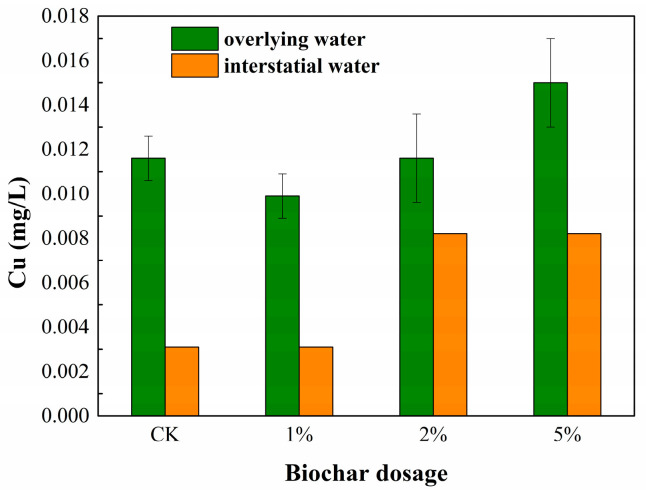
Cu concentration in the overlying water and interstitial water. The bars represent the standard deviations of the means (n = 3). The same below.

The Cu concentration in the overlying water and interstitial water are shown in Figure 1. The Cu concentration in the overlying water was extremely low, although the detected values of some treatments were higher than those of the CK treatment during the incubation process. The Cu concentration was still ≤ 0.015 mg/L, which is far less than the limit value of 1.0 mg/L stipulated in the Chinese Sanitary Standard for Drinking Water (GB 5749-2022) and the recommended value of 2.5 mg/L for drinking water stipulated by the World Health Organization (WHO) [34]. The results indicated that the addition of a certain amount of BC into the sediment did not cause significant Cu pollution in the overlying water.

The Cu concentration in the interstitial water after 90 days of incubation indicated that the Cu concentration of the 2% and 5% treatments reached 0.008 mg/L, which was higher than that of 0.003 mg/L of the CK treatment. However, the Cu concentration in the interstitial water was still at a very low level, and the Cu concentration in the interstitial water was not higher than that in the overlying water after 90 days of incubation. The above results demonstrated that Cu was primarily retained in the solid phase of the sediment.

Cd was not detected in all of the overlying water samples and the interstitial water samples during incubation. The results showed that the BC immobilized the heavy metal Cd in the sediment, or at least the BC application did not cause the release of Cd from the sediment to the water.

### 3.2. Sediment pH 

**Figure 2:**
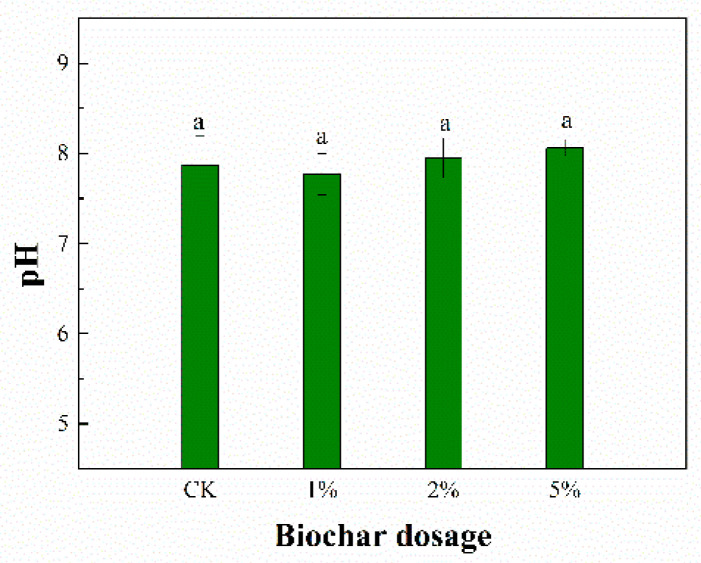
Sediment pH after the BC treatment. The different letters indicate significant differences at *p* < 0.05. The same below.

The pH value was detected as a basic parameter to analyze the characteristics of the sediment after the BC treatment. The pH value ranged from 7.8 to 8.1 (Figure 2). Compared with the CK treatment (pH 7.9), the pH value increased after 90 days of incubation, except for a slight decrease with the 1% treatment (pH 7.8). This pH increase can be attributed to alkaline substances (oxides, hydroxides, and carbonates) in the BC produced from base cations (primarily Ca, Mg, K, and Na) in the biomass [35]. At the initial stage of incubation, the addition of BC and the ammoniation of nitrogen-containing substances can generally increase the pH. It is well-accepted that a higher pH favors metal precipitation and simultaneously decreases metal solubility. Therefore, the result indicated that BC was beneficial for the immobilization of the Cu and Cd heavy metals after 90 days of incubation.

### 3.3. TCLP Leachability of Cu and Cd in Sediment 

**Figure 3:**
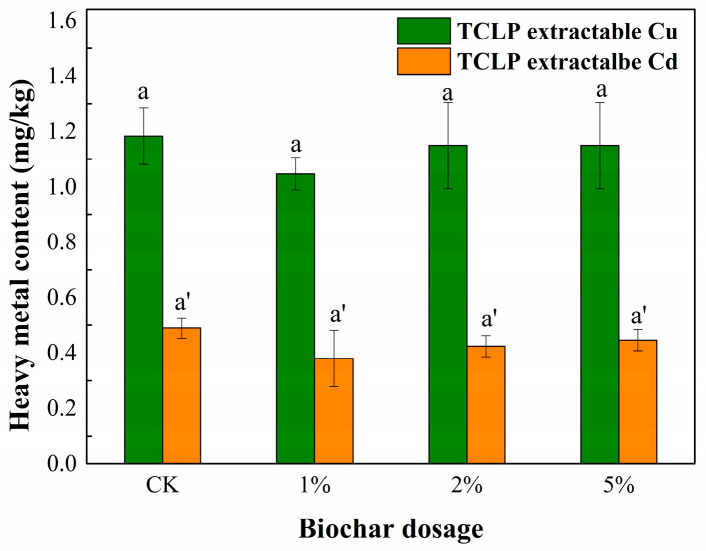
Concentration of the TCLP-extractable Cu and Cd in sediment after BC treatment.

The effect of BC on Cu and Cd immobilization was evaluated by determining their availability based on the TCLP. The TCLP contained extractable Cu and Cd in the sediment/solid system, as shown in Figure 3. The concentration of the TCLP-extractable Cu of the CK treatment was 1.18 ± 0.10 mg/kg, and the concentrations of the 1%, 2%, and 5% treatments were 1.05 ± 0.06 mg/kg, 1.15 ± 0.16 mg/kg, and 1.15 ± 0.16 mg/kg, respectively. A similar phenomenon of the concentration of the TCLP-extractable Cd was observed. The concentration of the TCLP-extractable Cd of the CK treatment was 0.49 ± 0.05 mg/kg, and the concentrations of the 1%, 2%, and 5% treatments were 0.38 ± 0.05 mg/kg, 0.42 ± 0.09 mg/kg, and 0.45 ± 0.05 mg/kg, respectively. The BC application facilitated the immobility of Cu and Cd, although BC contains a certain amount of Cu. The decrease in the concentration of the TCLP-extractable heavy metals could have been attributed to the BC’s reactive functional groups, such as carboxyl and hydroxyl groups, as shown in the FTIR spectrum. However, it is worth noting that the decrease was not significant, which means that the immobilization ability was limited.

### 3.4. Fraction of Cu and Cd in Sediment

**Figure 4:**
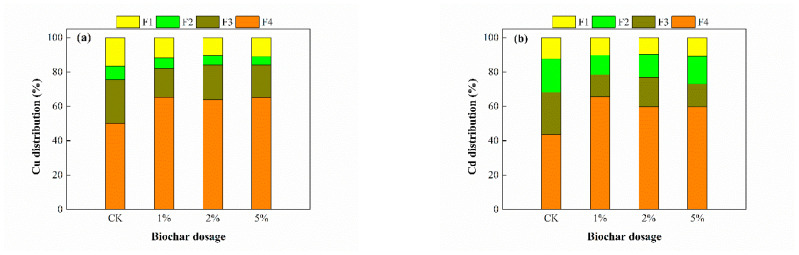
Fraction distribution of (**a**) Cu and (**b**) Cd determined using the BCR sequential extraction in sediments after BC treatment: acid-soluble fraction (F1), reducible fraction (F2), oxidizable fraction (F3), and residual fraction (F4).

The fraction distribution of Cu and Cd estimated using the BCR procedure in different treated sediment/solid systems is shown in Figure 4. In the treated sediment/solid system, Cu primarily existed in the residual fraction and oxidizable fraction, and the sum of the two fractions increased from 75.49% in the CK treatment to 81.91%, 84.03%, and 84.07% in the 1%, 2%, and 5% treatments, respectively (Figure 4a). With an increase in the BC dosage, the proportion of the acid-extractable fraction and oxidizable fraction correspondingly decreased. However, the phenomenon may have been partly due to the high residual fraction of the Cu in the BC, which was as high as 87.5% of the total Cu.

To clarify the effect of the Cu content in the BC, the fraction distribution of the Cu in the “sediment + BC” system before and after BC treatment was compared and is shown in Table 3. It can be seen from Table 3 that the Cu fraction changed before and after the treatment. The residual fraction significantly increased in the 1% and 2% treatments from 43.83% and 52.28% to 65.36% and 63.78%, respectively. The acid-soluble fraction, reducible fraction, and oxidizable fraction decreased. The residual fraction and oxidizable fraction in the 5% treatment slightly increased from 65.22% and 17.26% to 65.30% and 18.77%, respectively, and the acid-soluble fraction and reducible fraction correspondingly decreased. The results indicated that the Cu in the “sediment + BC” system was preferentially transformed into a more stable species.

Figure 4b shows that the Cd in the treated sediment primarily existed in the residual, oxidizable, and reducible fractions. The residual fraction showed an overall increasing trend after the BC treatment, from 43.6% in the CK treatment to 59.6–65.6%, and the other three fractions of Cd correspondingly decreased. Since the BC itself in this study did not contain Cd, it can be concluded that the BC application played a promoting role in the transformation of Cd to a more stable speciation, which is consistent with the accepted application of other materials to the remediation of metal-contaminated sediments [32]. Considering that the TCLP-extractable Cd (Figure 3) showed a decreasing trend, we can infer that the BC application contributed a certain amount of Cu to the sediment and was beneficial for Cd immobilization.

### 3.5. Enzyme Activities

**Figure 5:**
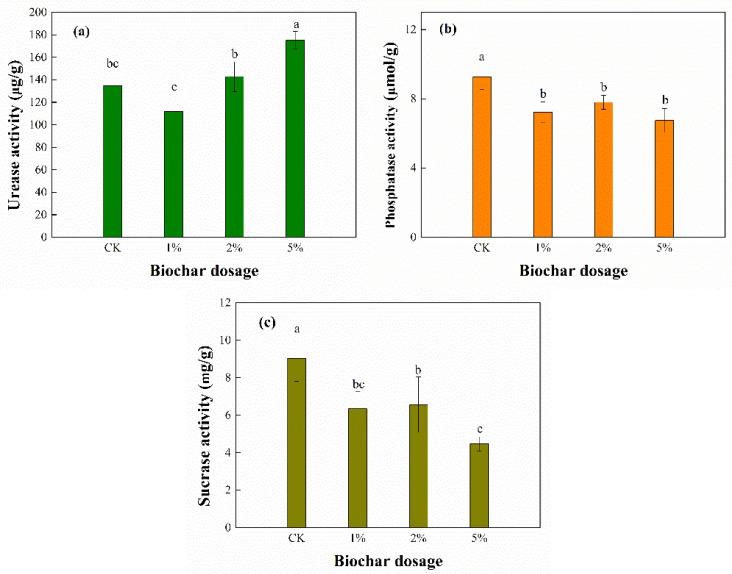
Effects of BC on (**a**) urease, (**b**) alkaline phosphatase, and (**c**) invertase activities in sediment.

Urease, alkaline phosphatase, and invertase activities were assayed to assess the impact of BC containing a certain amount of Cu on sediment microbes because enzymatic activities can directly address the biological availability and toxicity of heavy metals [32]. According to Figure 5a, urease activity increased as the BC dosage amount increased, except for in the 1% treatment. However, the decrease for the 1% treatment was not significant compared to that of the CK treatment. In particular, the urease activity of the 5% treatment (175.1 ± 7.9 μg/g) showed a significant increase and was 1.3-fold higher than that of the CK treatment (134.9 ± 13.1 μg/g). This indicated that there was a significant effect of BC on the urease activity.

The change in the urease activity might be explained by the combined effects of the BC itself and the extractable fraction of heavy metals. Previous studies discovered that BC could increase the activity of specific enzymes related to N utilization in soil [36] or that the nitrogen transformation related to urease activity was promoted by biochar [37]. In addition to BC itself, the changes in the extractable fraction of heavy metals could also explain the variations in urease activity. Huang et al. [31] found that there were significant negative correlations between heavy metals and urease activity. In this study, the extractable fraction of Cu and Cd decreased overall after BC treatment, which could have a positive effect on urease enzyme activity.

Figure 5b,c show that BC had a negative effect on the alkaline phosphatase and invertase activity. Both the alkaline phosphatase and invertase of all the BC treatments significantly decreased compared to those of the CK treatment, even at a low BC dosage. The alkaline phosphatase and invertase activity decreased from 9.26 ± 0.71 μmol/g and 9.03 ± 1.22 mg/g in the CK treatment to 7.22 ± 0.60 μmol/g and 6.34 ± 0.91 mg/g in the 1% treatment, respectively. In addition, the enzyme activities decreased with an increase in the BC dosage on the whole, although some of the differences between the different BC dosages were not significant.

Decreases in the alkaline phosphatase and invertase activity were also found with a high BC concentration [36,38]. The potential reasons included the following: (i) adsorption of enzymes or substrates on the BC that could impede the catalytic ability of enzymes such as β-xylosidase, lipase, and leucine aminopeptidase in sediment [36]; (ii) BC had negative effects on microbial behavior, such as material cell–cell communication and signal delivery in the microbic system [39]; (iii) the increase in pH value caused by the BC addition might be another explanation. A pH increase was observed in this study, and this was consistent with previous studies [31,40]. Another possible explanation for the decrease in alkaline phosphatase and invertase activity in this study is the inhibitory effect of heavy metals. Heavy metals typically have an inhibitory effect on microbial enzyme activities. However, the BC application facilitated Cu and Cd immobilization (Figure 3), which means the decrease in these two enzyme activities was not due to heavy metal inhibition. Therefore, the decrease in alkaline phosphatase and invertase activity could be attributed to the above three reasons associated with the BC itself.

### 3.6. Bacterial and Fungal Community Structure

The microbial community can reflect sediment quality. To further elucidate the effect of BC on indigenous microbes, the diversity indices (Shannon and Simpson) and richness index (Chao1) of the CK and the 2% and 5% BC treatments were calculated for bacteria and fungi based on the taxonomic information. From Table 4, for bacteria, the Chao1 indices of both the 2% and 5% treatments were higher than those of the CK treatment, but the Chao1 index of the 5% treatment was lower than that of the 2% treatment. Both the Simpson index and Shannon index showed the following ranking: 2% treatment > CK treatment > 5% treatment. By combining the above three indices, it was concluded that a lower BC dosage had an enhancing effect on bacterial abundance and diversity, while a higher BC dosage may have had a negative effect on diversity.

For fungi, the Chao1 estimator showed the following ranking: 2% treatment > CK treatment > 5% treatment. This indicated that a lower BC addition amount was beneficial for increasing the abundance of the fungal community, while a higher addition amount might have had a negative effect on the abundance. Both the Simpson index and Shannon index showed the following ranking: 2% treatment > 5% treatment > CK treatment. This indicated that the BC addition had a positive effect on the fungal community diversity, but a higher addition might have had a negative effect on the diversity.

**Figure 6:**
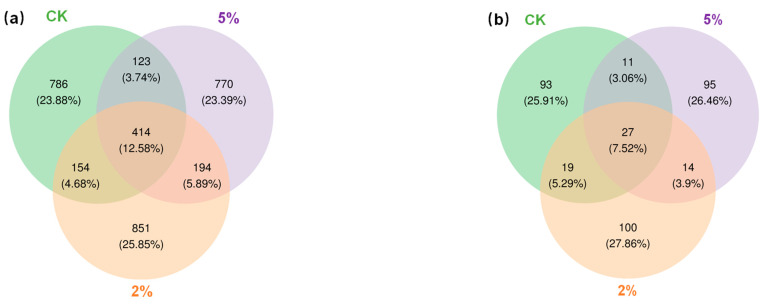
Venn diagrams of the sediment bacterial (**a**) and fungal (**b**) communities with the BC treatment.

The bacterial and fungal community variabilities are shown in Figure 6. From Figure 6a, it can be seen that the total bacterial OTUs in the sediments were 3292, and the common OTUs were 414. The OTU numbers showed the following ranking: 2% treatment > 5% treatment > CK treatment; and the specific bacterial OTUs of the CK treatment, 2% treatment, and 5% treatment were 786, 851, and 770, accounting for 23.88%, 25.85%, and 23.39% of the total OTUs, respectively. The total number of fungal OTUs was 359 (Figure 6b), and the number of common OTUs was 27. The OTU numbers showed the following ranking; 2% treatment > CK treatment > 5% treatment; and the specific fungal OTUs of the CK treatment, 2% treatment, and 5% treatment were 93, 100, and 95, accounting for 25.91%, 27.86%, and 26.46% of the total OTUs, respectively.

**Figure 7:**
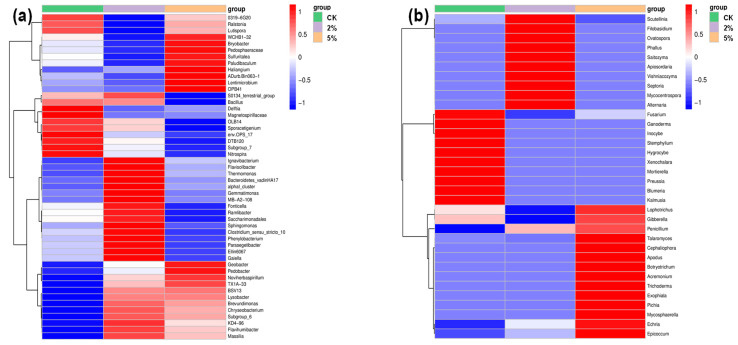
Hierarchically clustered heat map analysis of (**a**) the top 50 bacterial taxa and (**b**) the top 34 fungal taxa (relative abundances at the genus level) found in the BC-treated sediments.

To further compare the differences in the bacterial and fungal communities among the different treatments, a hierarchically clustered heat map analysis of the highly represented bacterial and fungal taxa (at the genus level) was conducted. Figure 7 shows the responses of the top 50 bacterial genera and 34 fungal genera to the BC treatment in the sediment slurries at the end of the 90-day experiment.

Figure 7a shows that *Delfita*, *Magnetospirillaceae*, *env.OPS_17*, *DTB120*, *Subgroup_7*, and *Nitrospira* were the most dominant bacterial genera in the non-treated sediment. After treatment with different BC dosages, the original strains of the dominant species disappeared, and some new dominants were found. With the 2% BC addition, *Ignavibacterium*, *Flavisolibacter*, *Thermomonas*, *Bacteroidetes_vadinHA17*, *Alphal_cluster*, *Gemmatimonas*, *MB-A2-108*, *Fonticella*, *Ramlibacter*, *Saccharimonadales*, *Sphingomonas*, *Clostridium_sensu+stricto_10*, *Phenylobacterium*, *Parasegetibacter*, *Ellin6067, Gaiella*, and *KD4-96* became dominant. In addition, *WCHB1-32*, *Bryobacter*, *Pedosphaeraceae*, *Sulfuritalea*, *Paludibaculum*, *Haliangium*, *ADurb.Bin063-1*, *Lentimicrobium*, *OPB41*, *Geobacter*, and *Pedobacter* became dominant, and new genera appeared in the 5% BC treatment.

A similar fungal genera change was observed, as shown in Figure 7b. A total of 10 genera, *Fusarium*, *Ganoderma*, *Inocybe*, *Stemphylium*, *Hygrocybe*, *Xenochalara*, *Mortierella*, *Preussia*, *Blumeria*, and *Kalmusia*, were the most dominant bacterial genera in the non-treated sediment. After the BC treatment, 10 (*Scutellinia*, *Filobasidium*, *Ovatospora*, *Phallus*, *Saitozyma*, *Apiosordaria*, *Vishniacozyma*, *Septoria*, *Mycocentrospora*, and *Alternaria*) and 11 (*Talaromyces*, *Cephaliophora*, *Apodus*, *Botryotrichum*, *Acremonium*, *Trichoderma*, *Exophiala*, *Pichia*, *Mycosphaerella*, *Echria*, and *Epicoccum*) new genera became dominant in the 2% treatment and 5% treatment, respectively.

The variations in the diversity of bacterial and fungal communities might be explained by the direct and indirect interactions between BC and microbes [41]. The direct interactions are as follows [41]: (i) BC can change soil properties, e.g., the pH value might play a key role in microbial abundance. An increase in the soil pH by 0.2–0.3 units after BC application can significantly affect the soil microbial community [42]. In this study, compared with the CK treatment (pH 7.9), the pH values of the 2% and 5% treatments increased to 8.0 and 8.1, respectively, after 90 days of incubation, which sequentially affected the indigenous microbes in sediment. (ii) BC can act as a microbial shelter with pore structures and surfaces. (iii) BC can improve and maintain nutrients for microbial growth. (iv) Free radicals and VOCs on BC can be toxic to some microbes. The indirect interactions are as follows [41]: (i) adsorbing enzyme molecules and influencing enzyme activities and elemental cycles; (ii) adsorbing and enhancing the hydrolysis of signaling molecules and, consequently, interrupting microbial communication and altering microbial community structure; (iii) enhancing the sorption and degradation of contaminants (e.g., TCLP-extractable Cu and Cd in this study), thus reducing the toxicity of contaminants to soil microbes.

The significant changes in the bacterial and fungal genera imply that the BC application derived from post-adsorbent may have induced an adverse impact on the sediment microbial community structure, especially in the high-BC-addition treatment; this could have caused a microbial activity change and, ultimately, resulted in changes in sediment function.

## 4. Conclusions

This study indicated that Cu and Cd concentrations were extremely low in the overlying water and interstitial water after sediment treatment with BC derived from the post-adsorbent. The pH value slightly increased, and the TCLP leachability of the Cu and Cd in the sediment decreased on the whole. Furthermore, Cu and Cd were preferentially transformed into more stable species. The findings highlighted the potential possibility of BC derived from post-adsorbent being used for Cu and Cd immobilization as a sediment amendment. However, the BC addition produced significant effects on the sediment microbial activity and community structure. With an increase in the BC amount, the urease activity increased, while the alkaline phosphatase and invertase activity decreased. In addition, significant changes in both bacterial and fungal genera were observed in sediments treated with BC. Therefore, the adverse effects should not be completely ignored once the BC is actually applied. These results represented an examination of the effects of the initial application of BC for sediment remediation. Further studies are required to reveal the long-term effects of BC application.

## Figures and Tables

**Table 1:** BC physicochemical properties.

Properties	Value
pH	8.3 ± 0.1
Yield (%)	20.2
Specific surface area (m^2^/g)	0.01
Elemental composition (%)	
C	74.85
H	1.24
N	1.25
Total Cu (mg/g)	2.76

**Table 2:** Sediment properties.

pH	Cd (mg/kg)	Cu (mg/kg)	Organic Content (%)	Total Nitrogen (g/kg)	Total Phosphorus (g/kg)
7.5 ± 0.1	3.98 ± 0.33	87.51 ± 0.5	5.1 ± 0.3	4.91 ± 0.13	0.39 ± 0.06

**Table 3:** Comparison of the Cu fraction in the “sediment + BC” system before and after treatment (percentage of total Cu).

Cu Fraction	1% Treatment		2% Treatment		5% Treatment	
	Before	After	Before	After	Before	After
Acid-soluble fraction (F1)	19.86	11.68	16.92	10.50	12.41	10.96
Reducible fraction (F2)	9.51	6.41	7.78	5.47	5.12	4.97
Oxidizable fraction (F3)	26.78	16.55	23.02	20.25	17.26	18.77
Residual fraction (F4)	43.83	65.36	52.28	63.78	65.22	65.3

**Table 4:** Bacterial and fungal alpha indices of sediment treated with BC.

Microbe	Treatment	OTU	Chao1	Simpson	Shannon	Coverage
Bacteria	CK	1477	1588.51	0.9610	7.6148	0.995
	2%	1613	1698.35	0.9814	8.3428	0.996
	5%	1501	1605.29	0.9314	7.4963	0.995
Fungi	CK	150	156.05	0.5210	2.5250	0.999
	2%	160	161.57	0.9391	5.1545	0.999
	5%	147	147.07	0.8225	4.1710	0.999

## Data Availability

Not applicable.

## Supplementary Materials

The following supporting information can be downloaded at https://www.mdpi.com/article/10.3390/toxics11080666/s1. Figure S1: SEM photograph (a) and FTIR spectrum (b) of BC.
