# Supplementary figures and images for: Biochar Derived from Post-Adsorbent for Immobilizing Cu and Cd in Sediment: The Effect on Heavy Metal Species and the Microbial Community Composition
Source: Toxics. 2023 Aug 2;11(8):666. doi: 10.3390/toxics11080666 (PMC10458644; doi:10.3390/toxics11080666)

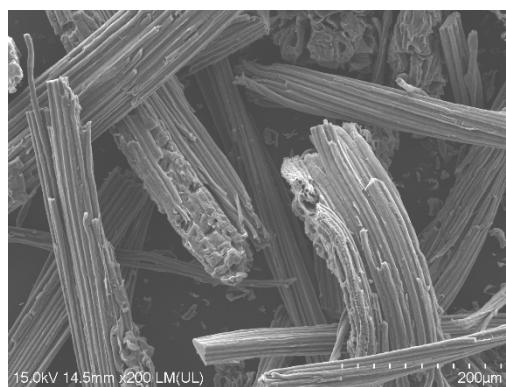

(a)

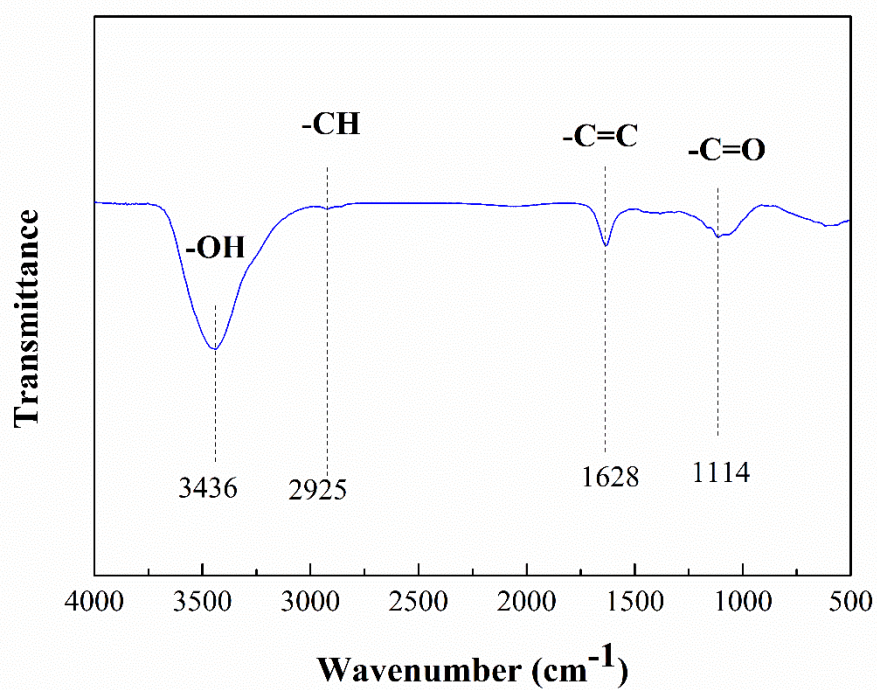

(b)

**Figure S1.** SEM photograph (a) and FTIR spectrum (b) of BC.

Supplement: Supplementary file 1 [file toxics-11-00666-s001.zip › toxics-2526129-supplementary.pdf]
